# Comparison of Volunteers’ Head Displacement with Computer Simulation—Crash Test with Low Speed of 20 km/h †

**DOI:** 10.3390/s22249720

**Published:** 2022-12-12

**Authors:** Damian Frej, Marek Jaśkiewicz

**Affiliations:** Department of Automotive Engineering and Transport, Kielce University of Technology, Avenue Tysiaclecia Państwa Polskiego 7, 25-314 Kielce, Poland

**Keywords:** computer simulation, crash test, dummy

## Abstract

Recently, the automotive industry has used simulation programs much more often than experimental research. Computer simulations are more and more often used due to the repeatability of simulation conditions and the possibility of making modifications in simulation objects. Experimental and simulation studies carried out are aimed at developing a model of a simulation dummy adapted to both frontal and rear crash tests, taking into account changes in the moment of resistance in individual joints. The main purpose of the article is to reproduce a real crash test at a low speed of 20 km/h in a simulation program. For this purpose, a series of experimental crash tests with the participation of volunteers was carried out, and then a crash test with a dummy was simulated in the MSC ADAMS program. The experimental studies involved 100 volunteers who were divided into three percentile groups (C5, C50, C95). With the help of force sensors and a high-speed camera, crash tests of volunteers were recorded. The collected data from the force sensors made it possible to map the force in the seat belts. For low-speed crash tests, the displacement and acceleration of individual body parts of the dummy and volunteers can be measured using vision systems. The article identified head displacements of volunteers in the TEMA program based on a video analysis of a crash test film with a frequency of up to 2500 frames per second. The displacement of the simulation dummy’s head in the MSC ADAMS program in the considered crash time interval from 0.0 to 0.4 s for all three percentile groups coincided with the head displacement of the volunteers during the experimental crash test.

## 1. Introduction

Globalization and the development of technology, in particular graphic programs, have permanently changed the approach to the design of new products, elements, security systems and complex car models [1,2,3].

The mathematical models being developed have become much cheaper than building a prototype. In addition, the current computer software allows for simulation in the assumed conditions and accurate measurement of the simulated element. It should be taken into account that in the case of the construction of a prototype, apart from the production costs, its development time was also extended [4,5,6].

Nowadays, even initial failures in model design do not incur high costs [4,7,8]. Computer programs make it possible to introduce corrections at every design stage, which is impossible when building a prototype [1,9,10]. The current computer technology has made mathematical models replace prototypes in most design and research works [11,12,13]. Mathematical models of products have become a fundamental manufacturing element in the 21st century. This does not mean that prototypes are no longer being built. Usually, after the completion of preliminary and simulation tests, as well as after careful selection of structural elements, its construction takes place. They are built to check computer simulations and for presentation purposes [14,15,16].

Without anthropometric dummies, the number of fatalities in road accidents would be much higher. It is to them that we owe a large contribution to the increase in the safety of motor vehicles [17,18,19]. There are currently about 3500 crash test dummies around the world [20,21,22,23]. Their design range includes genders, age groups and postures, from newborns through pregnant women to overweight old men [24,25,26]. They are specially adapted for different situations, for example, THOR for frontal impacts, SID for side impacts, BioRID II for rear impacts, CRABI for child seat tests and Hybrid III for pedestrian accidents. Each of them is designed for a specific crash test at a specific crash velocity [27,28,29]. None of the available models is used for low-speed crash tests and is not used in all types of crash tests [30,31,32].

The models of dummies used in crash tests consist of assemblies and elements resembling the human body. An ideal crash test dummy should have a simple design that does not require frequent calibration and, at the same time, be characterized by high durability and repeatability of results [33,34]. Currently, in the case of frontal collisions, the most advanced hybrid III and THOR dummy. Dummies from the Hybrid III family have a high compatibility index with the human body Research on the behavior of dummies during crash tests has led to the assessment of the head, face, chest, abdomen, lower and upper limb injuries, which are used by all countries around the world [34,35,36]. It should be noted that anthropometric dummies are dedicated to a specific type of test at a specific speed. None of the above dummies are dedicated to crash tests at low speeds, up to 20 km/h.

The crash test dummies are test objects equipped with a number of sensors. In the case of the Hybrid III dummy, its head is made of aluminum, and inside its head, there are sensors measuring the impact force and the acceleration of the head during the collision. There are force sensors in the cervical region that measure the forces that occur when bending and stretching the head and neck. The steel ribs of the Hybrid III dummy are equipped with sensors that measure the deviation of the chest during a collision. They show how great the risks of her injuries are as a result of the operation of the seat belts. In addition, the dummy’s abdomen and pelvis are equipped with force sensors to accurately diagnose the forces at which abdominal or pelvic injuries occur [37,38,39,40,41]. Sensors installed in the legs measure the deflection, pressure, and stresses that cause injuries to the tibia and fibula. There may be up to 200 sensors in the crash test dummies, depending on the type of dummy and the crash test. Moreover, the sensors are not only located in the anthropometric dummy but also in the vehicle intended for the crash test. Anthropometric crash test dummies are an invaluable object contributing to the improvement of vehicle safety. It should be noted that this is largely due to sensors that collect information about the forces, accelerations, and displacements of individual parts of the dummy’s body [42,43,44,45,46].

The authors of the paper [47,48,49] conducted experimental studies with the participation of volunteers. Volunteers participated in a series of low-speed rear crash tests of 6 to 8 km/h to determine the biomechanical and kinetic responses of the human body, taking into account various car seat headrest configurations. The authors showed differences in the displacement of the volunteers’ heads during the rear impact. The differences were caused by the dimensions of the human body, which are directly related to the sex of the volunteers. In the article [50,51], volunteers were used for low-speed head-on tests and low-speed side tests. The authors observed minimal differences in the displacement of individual parts of the human body in relation to male and female volunteers.

While reviewing, the authors of this paper noted that despite the differences in the displacement of individual parts of the human body due to the sex of the volunteers during low-speed crash tests. An anthropometric dummy dedicated exclusively to low-speed crash tests has not yet been developed.

## 2. Research Methodology

The article contains part of the work carried out in the laboratory of motor vehicles and tractors of the Kielce University of Technology. The purpose of the experimental crash tests with volunteers is to develop a new low-speed crash test physical dummy and to develop a low-speed simulation dummy. Research issues related to the protection of car passengers against the consequences of road collisions are classified as impact biomechanics. Therefore, in the methodology of my own research, two types of model analyzes are distinguished:

- Experimental “crash-test” research conducted with the participation of volunteers;
- Simulation studies performed in the MSC ADAMS program.

Simulation studies have many advantages over experimental crash-test studies. The main advantage is the low cost of building simulation models and the ease of conducting parametric research on virtual models.

## 3. The Course of Experimental Research

**Figure 1:**
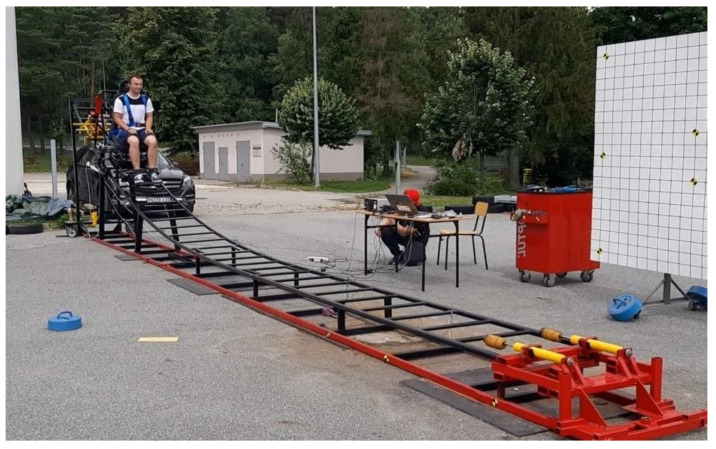
Test bench for low-speed crash tests.

Experimental tests with the use of volunteers were carried out on a test bench designed for crash tests at low speeds and measurements of force in the seat belts during a collision. The experimental crash test was carried out in the laboratory of Motor Vehicles and Tractors at the Kielce University of Technology. The crash test stand consists of a 10 m-long measuring track on which a platform with a car seat moves. At the end of the measuring track, there are two shock absorbers in a transverse position. The accelerated platform, together with the car seat to the assumed speed at the end of the measuring track, hits the shock absorbers simulating a collision with a solid obstacle. The platform with the car seat is pulled to the appropriate height of the measuring track with a winch located in the upper part of the measuring track. The platform, after being pulled to the appropriate height, is held by an electromagnet. Releasing the electromagnet initiates the measurement test. During this time, the platform, together with the car seat, moves down the test stand. Low-speed frontal crash tests involving volunteers were recorded using a high-speed Digital Phantom 310v camera that recorded the measurement at 2500 frames per second. The low-speed crash test bench is shown in Figure 1.

**Figure 2:**
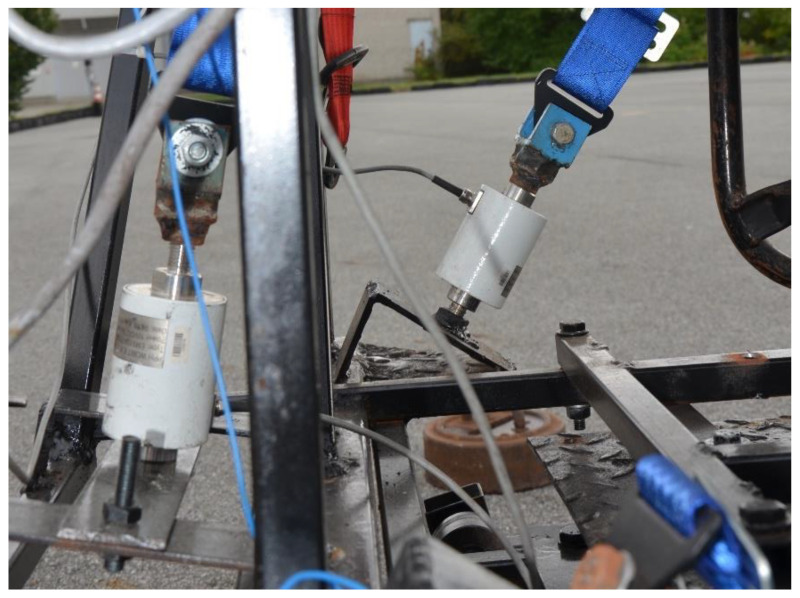
Force sensors in seat belts EMS 150.

The crash test stand enables the measurement of the force in the seat belts during the event test. There are two force sensors on the truck’s platform to which the seat belts are attached. The EMS 150 force sensors used to measure the force in seat belts are shown in Figure 2.

**Figure 3:**
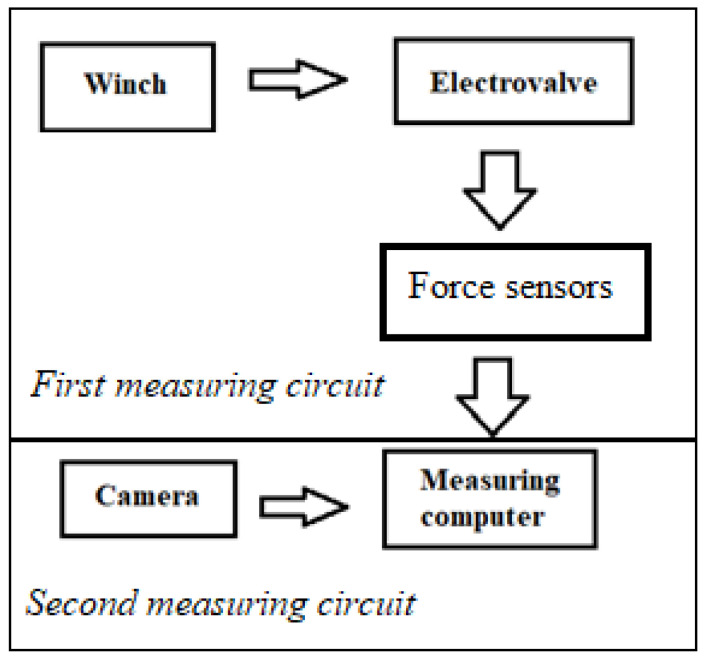
Diagram of the measuring station.

Figure 3 shows a diagram of the test stand, which consists of two independent circuits. The first one enables the recording of a crash test with the use of a high-speed Digital Phantom V310 camera. The image recorded by the camera is sent to the measuring computer and then displayed in the TEMA CLASSIC program. 

The experimental studies involved 100 volunteers who were assigned to the corresponding population percentile, and then the results were averaged. There were 20 volunteers in the C5 percentile group, 45 volunteers in the C50 group, and 35 volunteers in the C95 group. Examples of the dimensions of the person participating in the experiment are shown in Table 1. Each person participating in the crash test was measured and weighed. On the basis of 16 anthropometric parameters, volunteers were assigned to the appropriate percentile group. The anthropometric division of the subjects did not take into account gender because, with the low-speed crash test, the differences in the displacement of individual parts of the body were within 2%. The anthropometric data of the volunteers were compared with the current national standard PN-90/N-08000, which determines the dimensions of both men and women. 

## 4. The Course of Simulation Tests

The model of a physical-point crash test dummy is a set of interconnected bodies that are characterized by appropriate damping and stiffness. Each element has the appropriate shape and mass. All elements of the dummy’s structure are connected with each other by means of joints that reflect the range of human body movement. The simulation dummy model was made in the MSC Adams program. This program studies the dynamics of movement of individual parts of the body of a dummy. At the same time, it allows you to transform a rigid body into a flexible model using the finite element method. The program environment allows you to modify and change the parameters of individual elements of the dummy. In addition, it allows you to obtain information about the exact movement of all parts of the body of the dummy and then present them in a graphical way. The manikin was designed in the pattern of a hybrid III dummy representing a 50th-percentile male. It consists of 17 elements connected by joints. The manikin representing the C50 male was compared with the Hybrid III dummy during a crash test at 20 km/h.

**Figure 4:**
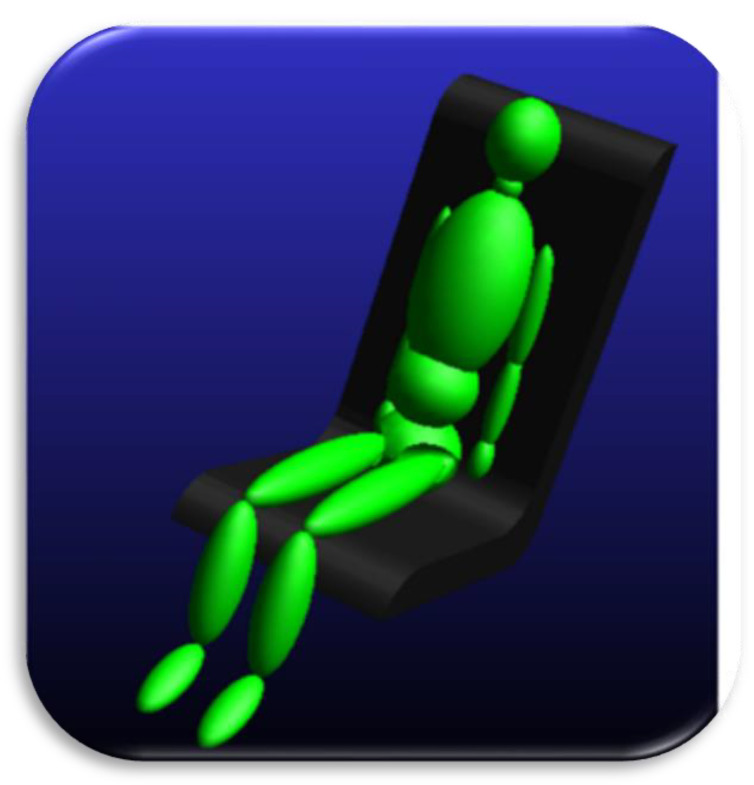
Anthropometric crash test dummy made in the ADAMS program.

The program environment makes it possible to modify and change the parameters of individual elements of the dummy. Therefore, the constructed simulation dummy may have any parameters (masses and dimensions) of individual elements of its body. The advantage of building an anthropometric manikin in the MSC Adams program is obtaining information on the exact movements of all parts of the manikin’s body immediately after computer simulation and the possibility of presenting this information in a graphical manner. An anthropometric dummy (representing a 50th centile male) designed for crash tests made in the ADAMS program is shown in Figure 4. During the construction of the anthropometric dummy, the following assumptions were made:

- system of rigid bodies;
- known dimensions, masses, and moments of inertia;
- a model in which the movement takes place on a three-dimensional plane;
- connection of solids by means of joints;
- the only input affecting the system is the initial speed vx (chair speed);
- belts and seats modeled on the basis of experimental research.

Table 2 lists the masses of individual body parts of the designed physical dummy representing a 50-cent man. Table 3 shows the lengths of individual sections of the designed dummy. The values of the joint angles in the joints of the designed dummy are presented in Table 4. The moments of inertia of the individual body parts of the simulation dummy were calculated using the Zatziorsky regression equations. Table 5 presents the moments of inertia of the simulation dummy representing the 50th percentile of the male population.

## 5. Initial Conditions of Computer Simulation

**Figure 5:**
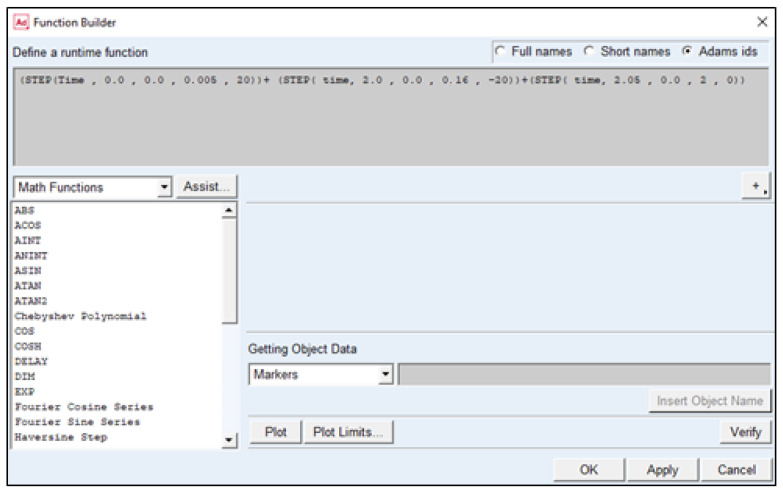
Motion function panel for simulation dummy.

In the MSC ADAMS program, the local XYZ coordinate system located at the base of the seat of the simulation dummy was introduced. This program made it possible in this adopted system to determine the displacements of individual body elements of the simulation dummy. The MSC ADAMS program allows you to enter the range of motion for individual joints located in the dummy or to introduce one movement for the entire object. Defining the movement for the entire facility is possible thanks to the built-in “step (time)” function. It was used to develop a crash test for low speed (20 km/h). This function forces the vehicle seat with the dummy to move in the direction of the *X*-axis until the speed of 20 km/h is reached, after which the speed drops sharply to 0 km/h, simulating a collision with an obstacle. Figure 5 shows the motion function panel for the simulation dummy, along with the equation used for the frontal impact at 20 km/h.

**Figure 6:**
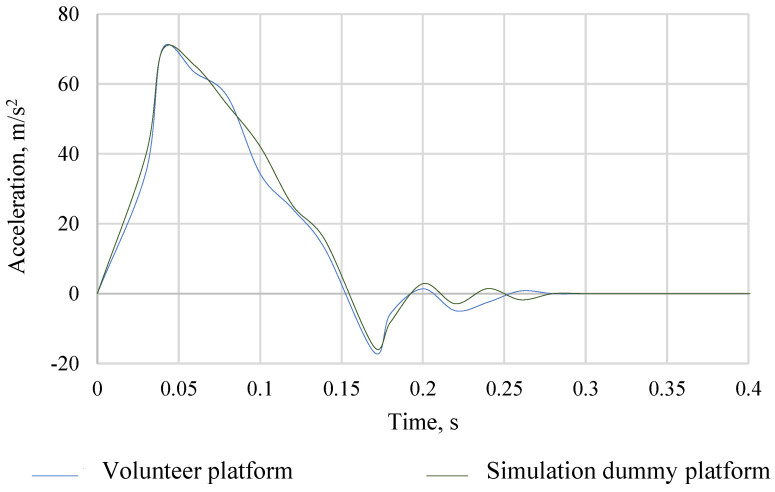
The course of acceleration of the stroller together with the vehicle seat during experimental studies.

**Figure 7:**
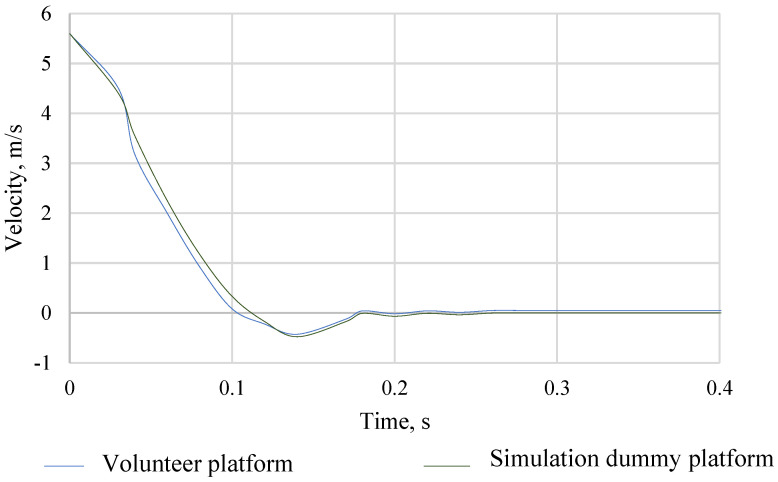
The course of the speed of the trolley during experimental studies.

Of great importance from the point of view of the correctness of simulation, tests are the repeatability of the acceleration pulse of the platform with the vehicle seat for the set crash speed. The acceleration of the platform, together with the vehicle seat for volunteers, was determined for each volunteer ride. In the case of crash tests in the MSC Adams program, the acceleration of the platform with a vehicle seat was mapped on the basis of real measurements with the participation of volunteers. The acceleration of the platform, together with the vehicle seat for the collision test with volunteers and the simulation dummy in the ADAMS program, is shown in Figure 6. The obtained results of mapping the acceleration of the volunteer platform with a vehicle seat in the ADAMS program were so satisfactory that it was possible to verify the model of the simulation dummy with volunteers. Then, by integrating, the speed waveform of the platform with the vehicle seat expressed in m/s was determined. The comparison of the speed course of the volunteer platform with the simulation dummy platform is shown in Figure 7. 

**Figure 8:**
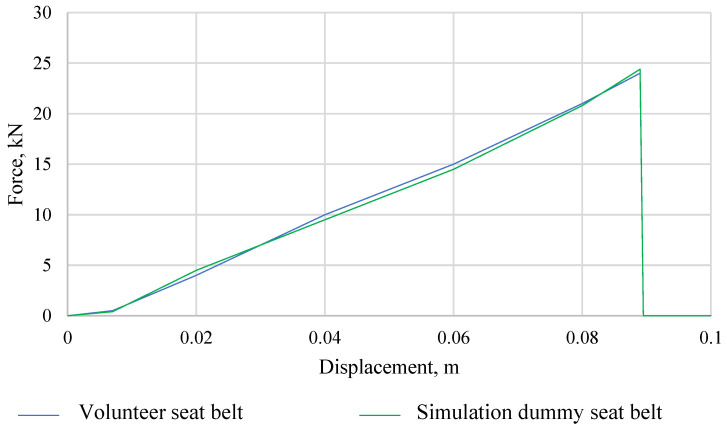
Comparison of the force characteristics in the seat belts during the volunteer crash test and the simulation dummy.

The second important parameter affecting the correctness of experimental and simulation tests is the use of identical seat belts. During the research, with the participation of volunteers, the force in the belts during the collision was measured. Then, the measured force in the belts during the collision was averaged on the basis of 10 crash tests with the participation of volunteers. The average value of the force in the belts during a low-speed collision was simulated in the MSC Adams program. Figure 8 shows a comparison of the force in the crash belts for the simulation dummy and the volunteers (average of 10 attempts). The obtained results of the force mapping in the belts during the collision in the ADAMS program were so satisfactory that it was possible to verify the simulation dummy model with volunteers.

## 6. Results

**Figure 9:**
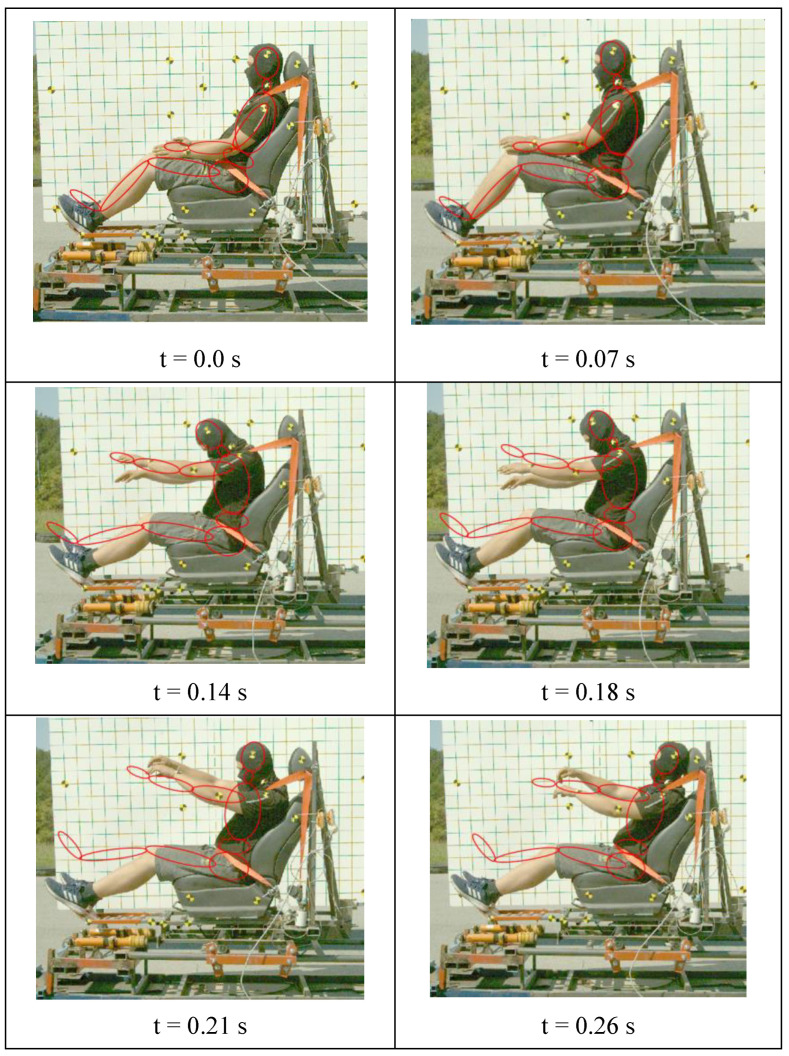
Frames at different times of the low-speed crash test with the participation of a volunteer representing the 50th percentile of the male population and the course of the simulation dummy crash test superimposed in the MSC ADAMS program.

Figure 9 presents examples of low-speed crash test frames with the participation of a volunteer representing the 50th percentile of the male population and the superimposed course of the crash test of a simulation dummy in the MSC ADAMS program. On its basis, comparable displacements of individual body parts of the volunteer with parts of the dummy can be observed. If the head, hands, and torso are displaced during the crash test at a speed of 20 km/h, the compliance of individual body parts with the simulation dummy is visible. In the case of the lower limbs, their greater displacement in 0.14 s of the crash test is due to the fact that the volunteers had support under their feet, which was not present during the crash test simulation.

In order to perform a computer simulation for the C5 and C95 percentile groups. The masses and dimensions of individual solids of the simulation dummy have been changed. The anthropometric dimensions of individual body parts of the C5 and C95 simulation dummies were selected in accordance with the human anthropometric atlas for the male population.

**Figure 10:**
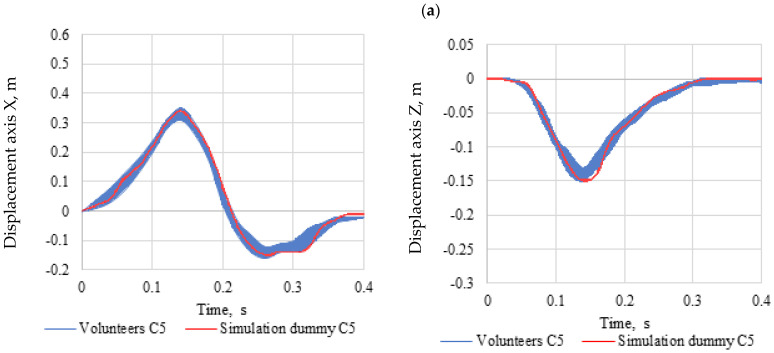

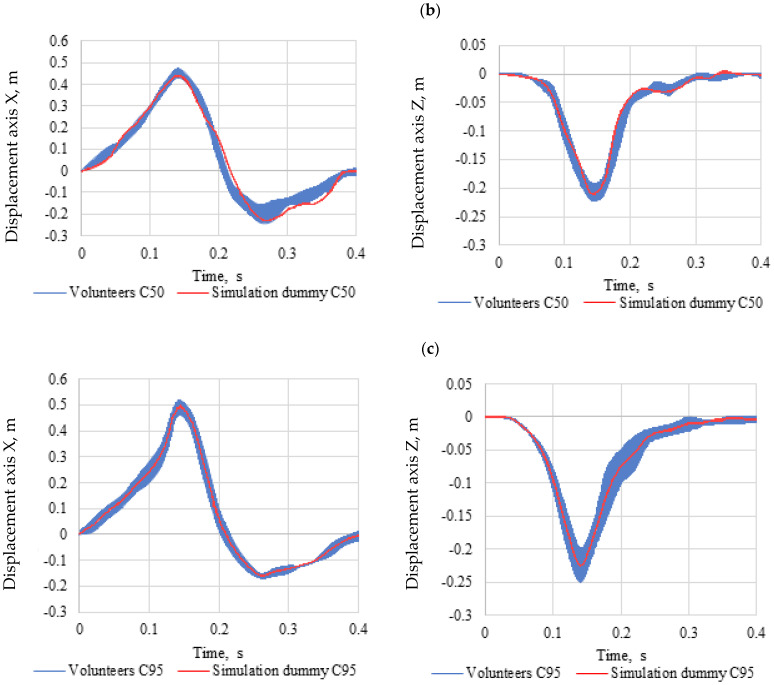
Volunteer head displacement, (**a**) C5, (**b**) C50, (**c**) C95.

Figure 10 shows a comparison of the volunteers’ head displacement with the simulation dummy made in the ADAMS program. The course of displacement of the head of volunteers and the simulation dummy was divided into three parts. The first part lasts from the moment of impact 0.0 s to 0.14 s. During this time, the head of the volunteers and the simulation dummy moves forward as much as possible. The second part of the crash test lasts from 0.14 s to 0.26 s. During this time, the heads of the volunteers and the simulation dummy move backwards as much as possible (the second phase of the head displacement of the volunteers contains negative values). The third phase lasts from 0.26 until it comes to a complete stop. The displacement of the volunteers’ heads and the simulation dummy was considered in the time from 0.0 s to 0.4 s. 

Undoubtedly, it should be noted that in the case of volunteers representing the C5 and C95 populations, the results obtained during the crash test in comparison with the results of the computer simulation are consistent at the level of 82%. In contrast, for volunteers representing the C50 population, the agreement was 88%. This difference was calculated based on the standard deviation determined for each pair of data over a specified period of time. The displacement of the head in the first phase of the collision is conditioned by the forces in the belts and in the second phase by the stiffness of the car seat. The car seat in the MSC ADAMS program consisted of a single block, while the car seat used in the experimental crash tests consisted of a seat, backrest, and headrest.

**Figure 11:**
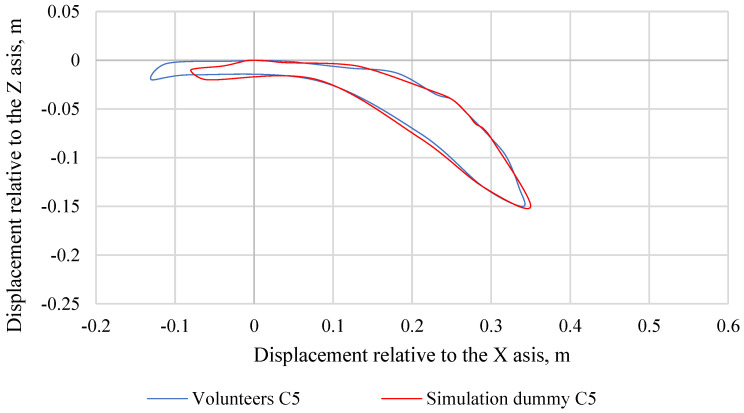
Comparison of head movement trajectories of a simulation dummy and volunteers representing the 5th percentile of the male population.

**Figure 12:**
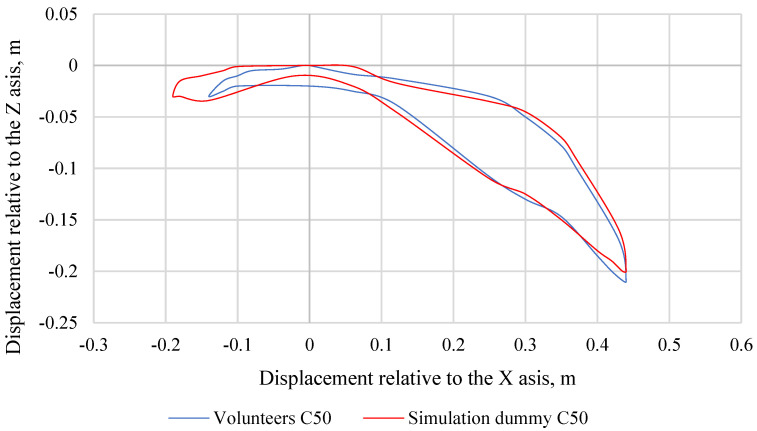
Comparison of head movement trajectories of a simulation dummy and volunteers representing the 50th percentile of the male population.

**Figure 13:**
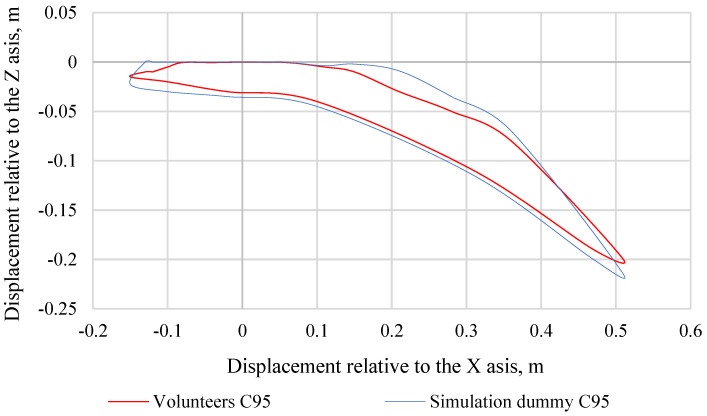
Comparison of head movement trajectories of a simulation dummy and volunteers representing the 95th percentile of the male population.

Making further comparisons between the simulation dummy and the volunteers, graphs were made showing the trajectories of the head movement. For volunteers, their head displacement range of motion corridors were averaged across each percentile group. Figure 11 shows the head movement trajectory of the C50 simulation dummy and C50 volunteers, while Figure 12 shows the head movement trajectory of the C5 simulation dummy and C5 volunteers. Figure 13 shows the head movement trajectory of the C95 simulation dummy and C95 volunteers. Differences in the values of the head movement trajectory during the crash test between the C50 simulation dummy and the C50 volunteers are insignificant and do not exceed 20%. This difference was calculated based on the standard deviation determined for each pair of data over a specific time period (0.00 s to 0.40 s). Differences in the head movement trajectory values during the crash test between the C5 simulation dummy and the C5 volunteers are insignificant and do not exceed 15%. Differences in the values of the head movement trajectory during the crash test between the C95 simulation dummy and the C95 volunteers do not exceed 18%. This difference was calculated based on the standard deviation determined for each pair of data over a specific time period (0.00 s to 0.40 s).

## 7. Discussion

Initial differences in the position of both the legs and upper limbs affect the movement of the head and, above all, changes in its position in relation to the seat. Determination of the head displacement is based on the identification of the position of the markers stuck in this case on the volunteer’s head. In all low-velocity crash tests, the displacement of the head of the volunteers is very large, ranging from 0.31 m to 0.49 m depending on the population percentile.

The head displacement in relation to the *X* axis in the case of volunteers representing the 5th percentile of the population in the first phase of the collision (0.14 s) ranges from 0.35 m to 0.38 m, and in the second phase of the collision (0.26 s) from 0.14 m to 0.17 m. In the case of people representing the 50 percentile of the population, in the first phase of the impact (0.14 s), the movement of the head in the direction of the *X*-axis ranges from 0.42 m to 0.49 m, and in the second phase of the impact (0.26 s) from 0.15 m to 0.23 m. Movement of the head in the direction of the *X* axis in the case of people representing the 95th percentile of the population in the first phase of the impact (0.14 s) ranges from 0.47 m to 0.55 m, and in the second phase of the collision (0.26 s) from 0.17 m to 0.21 m.

The head displacement in relation to the *Z* axis in the case of volunteers representing the 5th percentile of the population in the first phase of the impact (0.14 s) ranges from 0.12 m to 0.15 m. The displacement of the head in the direction of the *Z* axis, in the case of people representing 50th percentile of the population (14 s) ranges from 0.20 m to 0.24 m. The displacement of the head in the direction of the *Z* axis, in the case of people representing the 95th percentile of the population in the first phase of the impact (0.14 s) in the range from 0.22 m to 0.25 m. Movement of the head in the direction of the *Z* axis for all volunteers participating in the crash test in the second phase of the impact (0.26 s) ranged from 0.01 m to 0. 04 m.

The head displacements of the simulation dummy were superimposed on the designated corridors of the volunteers’ head space in the direction of the *X* and *Z* axes. Head displacement in the X and Z directions of the simulation dummy representing the 50th percentile of the population coincides 100% with the displacement of the head of the volunteers in the first phase of the impact. However, during 0.24 s of the crash test, the displacement of the simulation dummy’s head slightly exceeds the corridor of the volunteers’ head displacement. In the case of the simulation dummy representing the 5th percentile of the population, the head in both the first and the second phase of the impact in the direction of the *X*-axis coincides 100% with the head displacement of the volunteers.

## 8. Conclusions

Simulation programs play a major role in crash tests to increase the passive safety of passenger cars. In its simplest form, computer simulation is designed to reproduce phenomena that occur in the real world using mathematical models. They are defined and operated with the help of computer programs. In crash tests, experimental tests are associated with high costs. In contrast, simulation studies are much cheaper than experimental studies because they do not require a state of research. Modern simulation programs such as Dytran, Madymo or ADAMS are geared toward crash test simulations and data validation through experimental tests. 

In the case of crash tests at low speeds up to 20 km/h, the most sensitive element is the head and neck. Therefore, the authors of the article focused on the displacement of the volunteers’ heads and the simulation dummy during the low-speed crash test. Undoubtedly, it should be noted that the safest form of data recording during a low-speed crash test is the use of a high-speed camera. Crash tests involving volunteers were recorded at a frequency of 2.5 thousand frames per second. The measurement frequency used made it possible to compare the recorded crash tests with a computer simulation, which was also recorded at a frequency of 2.5 thousand frames per second.

The selected MSC ADAMS simulation program enabled the construction of a simulation dummy consisting of solids connected by joints that are available in the program. The program allows you to modify the entered parameters, such as mass, dimensions of individual body parts and the moment of resistance in the joints. Thanks to this, the simulation dummy model can be adapted to the dimensions of the appropriate percentile group. The simulation dummy made in the MSC ADAMS program was modeled in such a way as to reproduce the movements of volunteers during the crash test at a low speed of 20 km/h. Depending on the percentile group, the displacement of the head of the simulation dummy in relation to the volunteers in the considered period of time achieved compliance at the level of 80%. On the basis of simulation studies and experimental studies with the participation of volunteers, it should be noted that anthropometric dimensions affect the trajectories of head movement. The smallest displacement of the head in the direction of the *X* and *Z* axes occurred in the case of volunteers representing the 5th percentile of the population and the largest in the case of volunteers representing the 95th percentile of the population.

The next stage of work related to low-speed crash tests will be the construction of a physical anthropometric dummy. Satisfactory results of the probability of displacement of individual body parts of the simulation dummy with volunteers allowed for the initial validation of the dummy. The next step will be to compare the simulation dummy with the physical dummy in both the frontal crash test and the low-speed rear crash test. The creation of a physical dummy dedicated to a low-speed crash test will allow for an analysis of what happens to the frontal body during rear and frontal collision using one dummy.

Work on the physical construction of the simulation dummy involves the use of elements corresponding to the shape, mass, and dimensions of individual parts of the human body, as well as the use of special joints reflecting the range of motion of individual human joints. Crash tests in the automotive world concern the safety of passengers and the behavior of structures under the influence of dynamic impacts. Crash testing requires specific equipment and systems. It should be noted that it is not possible to conduct a crash test without an appropriate anthropometric dummy and data recording system.

## Figures and Tables

**Table 1:** Anthropometric dimensions of the volunteer.

Parameters	Values	Assignment to the Population Percentile
Mass [kg]	65	C50
Height [cm]	175	C50
Head circumference [cm]	59	C95
Torso length [cm]	60	C95
Chest circumference [cm]	95	C50
Arm circumference [cm]	37	C50
Arm length [cm]	33	C50
Forearm circumference [cm]	31	C50
Wrist circumference [cm]	20	C50
Wrist width [cm]	10	C50
Hand width [cm]	22	C50
Thigh circumference [cm]	57	C50
Circumference of the lower Leg [cm]	40	C50
Ankle circumference [cm]	26	C50
Foot length [cm]	24	C50
Volunteer qualified for C50 population		

**Table 2:** Masses of individual body parts of the simulation dummy.

LP	Simulation Dummy	
	Block Name	Mass, kg
1	forearm	4.04
2	arm	4.00
3	hand	0.54
4	shank	10.08
5	foot	0.80
6	thigh	11.98
7	neck	1.54
8	head	4.54
9	hips	11.35
10	chest	17.64
11	belly	12.19
(Σ)		78.70 kg

**Table 3:** Lengths of individual sections of the designed dummy.

LP	Episode	Simulation Dummy
		Length, mm
1	head (GZ)	161
2	neck (ZW)	124
3	chest + belly (WV)	443
4	hip (VH)	110
5	thigh (HK)	310
6	drumstick with foot (KS)	445
7	arm (BL)	255
8	forearm with hand (LN)	243

**Table 4:** Values of articular angles in the joints of the designed dummy.

Joint	Δφ	Δφ_min,_ Deg	Δφ_max,_ Deg
Z	φ_1_ − φ_2_	−10	55
W	φ_2_ − φ_3_	−35	5
V	φ_3_ − φ_4_	−85	30
H	φ_4_ − φ_5_	55	195
K	φ_6_ − φ_5_	−130	0
B	φ_7_ − φ_3_	−230	0
L	φ_8_ − φ_7_	0	150

**Table 5:** Moments of inertia of a simulation dummy representing the 50th percentile of the male population.

Body Parts	Unit	Simulation Dummy M50
moment of inertia of the neck	kg·m^2^	0.003
moment of inertia of the head	kg·m^2^	0.024
moment of inertia of the upper torso	kg·m^2^	0.248
moment of inertia of the middle part of the torso	kg·m^2^	0.198
moment of inertia of the lower torso	kg·m^2^	0.062
moment of inertia of the arm	kg·m^2^	0.023
moment of inertia of the forearm	kg·m^2^	0.007
moment of inertia of the hand	kg·m^2^	0.001
moment of inertia of the thigh	kg·m^2^	0.183
moment of inertia of the leg	kg·m^2^	0.093
moment of inertia of the foot	kg·m^2^	0.004

## Data Availability

Informed consent was obtained from all subjects involved in the study.
